# A quality assessment of Health Management Information System (HMIS) data for maternal and child health in Jimma Zone, Ethiopia

**DOI:** 10.1371/journal.pone.0213600

**Published:** 2019-03-11

**Authors:** Mariame Ouedraogo, Jaameeta Kurji, Lakew Abebe, Ronald Labonté, Sudhakar Morankar, Kunuz Haji Bedru, Gebeyehu Bulcha, Muluemebet Abera, Beth K. Potter, Marie-Hélène Roy-Gagnon, Manisha A. Kulkarni

**Affiliations:** 1 School of Epidemiology and Public Health, University of Ottawa, Ottawa, Ontario, Canada; 2 Department of Health Behavior and Society, Public Health Faculty, Jimma University, Jimma, Oromiya, Ethiopia; 3 Jimma Zonal Health Office, Jimma, Oromiya, Ethiopia; 4 Department of Population and Family Health, Public Health Faculty, Jimma University, Jimma, Oromiya, Ethiopia; Nathan S Kline Institute, UNITED STATES

## Abstract

Health management information system (HMIS) data are important for guiding the attainment of health targets in low- and middle-income countries. However, the quality of HMIS data is often poor. High-quality information is especially important for populations experiencing high burdens of disease and mortality, such as pregnant women, newborns, and children. The purpose of this study was to assess the quality of maternal and child health (MCH) data collected through the Ethiopian Ministry of Health’s HMIS in three districts of Jimma Zone, Oromiya Region, Ethiopia over a 12-month period from July 2014 to June 2015. Considering data quality constructs from the World Health Organization’s data quality report card, we appraised the completeness, timeliness, and internal consistency of eight key MCH indicators collected for all the primary health care units (PHCUs) located within three districts of Jimma Zone (Gomma, Kersa and Seka Chekorsa). We further evaluated the agreement between MCH service coverage estimates from the HMIS and estimates obtained from a population-based cross-sectional survey conducted with 3,784 women who were pregnant in the year preceding the survey, using Pearson correlation coefficients, intraclass correlation coefficients (ICC), and Bland-Altman plots. We found that the completeness and timeliness of facility reporting were highest in Gomma (75% and 70%, respectively) and lowest in Kersa (34% and 32%, respectively), and observed very few zero/missing values and moderate/extreme outliers for each MCH indicator. We found that the reporting of MCH indicators improved over time for all PHCUs, however the internal consistency between MCH indicators was low for several PHCUs. We found poor agreement between MCH estimates obtained from the HMIS and the survey, indicating that the HMIS may over-report the coverage of key MCH services, namely, antenatal care, skilled birth attendance and postnatal care. The quality of MCH data within the HMIS at the zonal level in Jimma, Ethiopia, could be improved to inform MCH research and programmatic efforts.

## Introduction

As a comprehensive, disaggregated and comparable source of information about health services in low and middle-income countries, health management information systems (HMIS) have the strong potential to act as the cornerstone for effective policy decision making[1,2]. Yet, numerous issues exist related to the completeness, timeliness, and accuracy of HMIS data [3–13], resulting in the tendency for countries and global health initiatives to rely on indicators measured through population-based surveys, such as the Demographic Health Survey and Multiple Indicator Cluster Survey. These surveys, however, do not provide continuous estimates of population-level indicators given their cross-sectional nature [2,11].

As part of the Federal Ministry of Health (FMoH) “*One Plan*, *One Budget & One Report*” policy, Ethiopia contracted the consulting firm John Snow, Inc in the years 2006–2007 to assess and redesign its HMIS. The aim of this effort was to improve the management and optimum use of resources for making timely decisions and promoting effective health care system delivery [14]. This evaluation found that the burden of HMIS data management was high, different partners were independently gathering the same information, standardized indicator definitions among the different partners and regions were lacking, data were of poor quality, and the use of HMIS information was weak and highly centralized at the highest levels of the reporting system [14,15]. Given the paper-based nature of the HMIS at that time, the project also resulted in the design and deployment of an electronic HMIS in health facilities nationwide [14]. Following the 2006 reform, another assessment in 2009–2010 highlighted that the HMIS remained “cumbersome and fragmented” [14], with persisting poor quality of data and inadequate skills for collecting, analyzing and interpreting the information among the health care staff at the lowest levels of the health system [14].

More recent work has found that Ethiopia’s HMIS remains particularly weak at the primary health care unit (PHCU) and district health office levels [15–20]. A study conducted by Teklegiorgis et al. in Eastern Ethiopia indicated that completeness and timeliness of data reporting were below national targets, with rates of 82% and 77%, respectively [19]. In the same study, 65% and 42% of health facilities used the information gathered for decision-making and for observing trends in service delivery, respectively [19]. In another study conducted in Jimma Zone, timeliness was determined to be poor with fewer than 50% of health posts, health centres and district health offices reporting within their given deadline [17]. Thirty-eight percent of health workers recognized that their reports and registration books may contain inconsistencies [17].

To our knowledge, no study in Ethiopia has focused specifically on the quality of maternal and child health (MCH) data collected within the HMIS. Recognizing the importance of collecting quality health data for populations facing a high burden of morbidity and mortality, such as pregnant women, newborns, and children, we assessed the quality of MCH data collected through Ethiopia’s HMIS over a 12-month time period in three districts of Jimma Zone, Southwest Ethiopia. To this end, we used data quality dimensions proposed by the World Health Organization (WHO) and MCH indicator estimates from a cross-sectional household survey conducted within the same time frame.

## Materials and methods

### Study setting

Jimma Zone is located in Oromiya region, Southwest Ethiopia, approximately seven hours from Addis Ababa, Ethiopia’s capital. The total population of this Zone is estimated at approximately 3.3 million inhabitants. Jimma Zone is further divided into 21 *Woredas* or districts. Three of the districts in Jimma Zone were selected for a large cluster-randomized trial designed to address barriers to safe motherhood (ClinicalTrials.gov identifier: NCT03299491): Gomma, Kersa, and Seka Chekorsa. Ten PHCUs are located in Gomma, nine in Seka Chekorsa and seven in Kersa, resulting in a total of 26 PHCUs. A PHCU consists of one health centre associated with five health posts. Each health post is managed by two government-employed health extension workers, trained in the delivery of primary care to communities within their catchment area.

### Data sources

#### Health management information data

The primary level of health service delivery and data collection in Ethiopia occurs in PHCUs. The compiled data from PHCUs are forwarded to and reviewed by the district health offices before being submitted to the zonal health office, where the data are entered into the electronic HMIS and made accessible to regional and national levels policy-makers [21].

For our study, we consulted with the Jimma Zonal Health Office and extracted electronic monthly service reports for the 2015 fiscal year (i.e. July 2014 to June 2015) for Gomma, Kersa and Seka Chekorsa district health offices and their respective PHCUs. Monthly reports from the two previous fiscal years (i.e. July 2012-June 2013 and July 2013-June 2014) were also retrieved to assess internal consistency.

#### Cross-sectional survey data

As part of the baseline evaluation for the above-mentioned cluster-randomized trial designed to address barriers to safe motherhood in Jimma Zone, a community-based population-representative cross-sectional survey was performed with women aged 15–49 years from the three districts, who had had a birth outcome (live birth / stillbirth / miscarriage / abortion) within the same time frame as the HMIS extracted reports. Results from this survey are the subject of a separate publication and are not presented herein. To attain the sample size for the trial, a two-stage sampling strategy was used. Twenty-four PHCUs or clusters were first randomly selected from the 26 available in the three study districts. From each PHCU catchment area, 160 eligible women were then randomly selected from registration lists compiled by volunteer community health workers in each village or *kebele*. A total of 3,784 women provided information on their past use of maternal and child health services as well as on their experiences when they were pregnant, during childbirth, and after delivery (see survey questions in S1 Appendix). After obtaining informed consent, face-to-face surveys were conducted at the women’s households by trained interviewers. For our purposes, we only consider those participants who reported attending any of the MCH services in one of the PHCUs found in the three districts. This resulted in the exclusion of 2.5% of the total sample of women who attended a health facility located in another district.

### World Health Organization data quality report card

Recognizing the need for developing countries to regularly evaluate the quality of their routine health information system, the WHO developed several tools to assist in assessing common data quality dimensions, including the Data Quality Report Card (DQRC), which we used in this study. The DQRC considers several data quality dimensions and represents a relatively easy and quick quantitative method to identify inaccuracies and inconsistencies in HMIS data [22]. Four dimensions of data quality are included in the DQRC: completeness of reporting; the internal consistency of reported data; the external consistency of population data; and, external consistency of coverage rates [23]. These four dimensions are further sub-divided into specific elements or indicators (Table 1). Given the unavailability of appropriate district-level census data, we did not assess the external consistency of population data.

**Table 1:** Data quality dimensions and indicators defined in the World Health Organization data quality report card.

Indicator	Definition^a^
**Dimension 1: Completeness of reporting**	
Completeness of health facility reporting	The percentage of expected monthly health post and health centre reports that were received by the three district health offices.
Timeliness of health facility reporting	The percentage of monthly reports from health posts and health centres that were received on time by their respective district, according to the fixed deadline.
Completeness of MCH indicator data	The number of missing and zero^b^ values for each MCH indicator in each PHCU and district service report.
**Dimension 2: Internal Consistency of reported data**	
Accuracy of event reporting: identification of moderate and extreme outliers	The number of moderate and extreme outliers for each MCH indicator in each PHCU and district service report. Moderate outliers were defined as values that were at least ± two standard deviations from the average value for a specific indicator for a given district at a specified time. Values were considered extreme outliers when they were at least ± three standard deviations from the average value.
Consistency over time	Consistency of the number of events for a MCH indicator in the year of analysis compared with the average number of events reported for the same indicator for the two previous years combined expressed as a ratio (i.e. number of events in current year divided by average number of events in two previous years).
Internal consistency between indicators	Consistency between the number of events reported for two indicators expected to correlate. MCH indicators expected to correlate include: ANC1 and DTP1^c^ ANC1 and ANC4^d^ DTP1 and DTP3^e^
**Dimension 3: External consistency of population data**	
Consistency of Population Projections^f^	Consistency between official country projection for live births and United Nations population projection for the year of interest.
Consistency of denominator	Comparison of district-level official estimates for pregnant women and children under 1 year of age with alternate estimates for pregnant women and children under 1 year derived from an alternate source (e.g. household-based survey).
**Dimension 4: External consistency of coverage rate**	The level of discordance between data collected through the HMIS and the estimates obtained from the recent survey conducted with women who had a birth outcome in the preceding year.

ANC1 –Antenatal Care First Visit; ANC4 –Antenatal Care Fourth Visit; DTP1 –Diphteria, Tetanus, Pertussis first dose; DTP3 –Diphteria, Tetanus, Pertussis third dose; MCH—Maternal and Child Health; PHCU—Primary Health Care Unit

^a^ Definitions adapted from the Data Quality Report Card (DQRC) guideline for this study

^b^ We only assessed MCH indicators for which no true zero values would be expected

^c^ Women who seek care during their pregnancy are also more likely to seek care for their children [23]

^d^ The number of ANC4 visits should either be approximately similar to or lower than the number of ANC1 visits recorded but never higher

^e^ The number of DTP3 doses should either be approximately similar to or lower than the number of DTP1 doses administered but never higher

^f^ This indicator cannot be calculated at the sub-national (i.e. district) level given the absence of UN population projections at that level.

### Maternal and child health indicators

In this data quality assessment, we examined several key maternal health services and immunization coverage indicators recommended by the WHO [23], including: antenatal care first (ANC1) and fourth (ANC4) visit coverage; deliveries attended by a skilled birth attendant (SBA) in health facilities; access to early postnatal care (PNC); and, infant receipt of Diphtheria, Tetanus, Pertussis vaccine first (DTP1) and third (DTP3) dose. We also included two additional indicators: malaria in pregnancy and stillbirth rate.

### Statistical analysis

We generated appropriate descriptive statistics for the first three dimensions of data quality according to the DQRC, using percentages, means, and standard deviation estimates. All analyses were performed using SAS statistical software version 9.4. (SAS Institute Inc., Cary, NC, USA).

To assess the fourth dimension of data quality (i.e. external consistency of coverage rates), we considered rigorous statistical tests that were not part of the DQRC. We generated scatter plots along with Pearson correlation coefficients and intraclass correlation coefficients to determine whether coverage rates from HMIS and survey data were linearly associated and the amount of variability between the two methods, respectively. We calculated ICCs using the Deyo’s method, as described by Szklo and Nieto [24]. Deyo’s method quantifies the variance of the difference between measurements taken by two scorers or readings (i.e. between-scorers/readings) as well as the variability within the repeated measurements recorded by each scorer (i.e. within-scorer). In this study, the ICC calculations took into account the variability between the two methods of MCH coverage rate estimation (i.e. HMIS vs. cross-sectional survey) and the variances reported within district by each method. We used Bland-Altman analysis, a standard method to assess agreement between two quantitative measurements, to estimate the concordance between MCH indicator values from the HMIS and the survey [25]. Given the number of data points in each district (i.e. ten, nine and seven PHCUs in Gomma, Seka Chekorsa and Kersa, respectively), adherence to the normal distribution might have compromised the results of the Bland and Altman analysis. We therefore estimated the limits of agreement using non-parametric methods [26]. Results using limits of agreement defined by the WHO are also available upon request.

## Ethics approval

Given low levels of literacy in the survey population, verbal informed consent was obtained all survey participants following verbal explanation of the contents of consent forms in the local language (Amharic or Afaan Oromo). Ethical clearance for this study and the verbal consent procedure was obtained from the Research Ethics and Integrity Board at the University of Ottawa and the Jimma University Institutional Review Board.

## Results

### Completeness of reporting

#### Completeness and timeliness of reporting

Data completeness was the highest in Gomma district with 76% of expected monthly health post and health centre reports received by the district health office, followed by 49% in Seka Chekorsa and 33% in Kersa (Table 2). Similar patterns were observed for timeliness of PHCU reporting. Gomma district health office received 71% of its expected PHCU reports on time, compared to 33% and 50% in Kersa and Seka Chekorsa, respectively.

**Table 2:** Completeness, timeliness and accuracy of reporting of HMIS data in the three districts in Jimma Zone, Ethiopia (2014–2015) based on an assessment of selected MCH indicators using the data quality report card.

	Gomma (%)	Kersa (%)	Seka Chekorsa (%)
**Completeness of reporting**	75.8	33.5	49.5
**Timeliness of reporting**	70.9	32.8	49.5
**Content completeness**	99.6	99.1	100.0
**Moderate Outliers**	4.3	4.7	3.1
**Extreme Outliers**	0.1	0.0	0.1

#### Content completeness

Of the submitted reports, Kersa had four (0.8%) missing indicator values, while Gomma only had three (0.4%) missing values all found within one PHCU. No zero or missing MCH indicator values were found in Seka Chekorsa reports (Table 2).

### Internal consistency of reported data

#### Accuracy of event reporting

Kersa had the largest proportion of moderate outliers, with 4.7% of the MCH indicator values being at least two standard deviations from their average value for the two preceding years, compared to 4.3% Gomma and 3.1% in Seka Chekorsa (Table 2). Very few extreme outliers were detected in the three districts.

#### Consistency over time

An increase in the uptake of MCH services over time was observed in all districts and in most PHCUs (Table 3). Postnatal care (PNC) showed the greatest increase in coverage, expressed as a ratio of the number of events in 2014–2015 relative to the average number of events in the two previous years. In contrast, ANC1, DTP1 and DTP3 coverage rates were relatively consistent over time in the three districts.

**Table 3:** Consistency over time ratios for HMIS data in three districts in Jimma Zone, Ethiopia (2012–2015) based on an assessment of selected MCH indicators using the data quality report card.

	Maternal and Child Health Indicators					
District	ANC1	ANC4	SBA	PNC	DTP1	DTP3
Gomma	1.0	1.9	1.5	4.8	0.9	0.9
Kersa	0.9	2.5	1.2	7.7	1.1	1.3
Seka Chekorsa	1.0	2.1	1.3	3.3	1.0	1.1

ANC1 –Antenatal care first visit; ANC4 –Antenatal care fourth visit; SBA- Skilled birth attendance; PNC—Postnatal care; DTP1 –Diphtheria, Tetanus, Pertussis first dose, DTP3 –Diphtheria, Tetanus, Pertussis third dose.

#### Internal consistency between indicators

Consistency between the number of ANC1 visits and number of DTP1 doses was low in Gomma, with a ratio of 0.58, suggesting that 42% more women attended an ANC1 visit than children receiving their first dose of DTP1 (Table 4). Similar trends were observed in Kersa and Seka Chekorsa, with 30% and 63% greater ANC1 coverage than DTP1 administration, respectively.

**Table 4:** Internal consistency of HMIS data in three districts in Jimma Zone, Ethiopia, (2014–2015) based on an assessment of selected MCH indicators using the data quality report card.

	Gomma	Kersa	Seka Chekorsa
**Ratio of DTP1 to ANC1**	0.58	0.70	0.37
**Number of PHCUs with DTP3/DTP1 above 1/Total number of PHCUs**	0/10	0/7	4/9
**Number of PHCUs with ANC4/ANC1 above 1/ Total number of PHCUs**	1/10	1/7	4/9

ANC1 –Antenatal care first visit; ANC4 –Antenatal care fourth visit; DTP1 –Diphtheria, Tetanus, Pertussis first dose, DTP3 –Diphtheria, Tetanus, Pertussis third dose; PHCU—Primary health care unit.

All PHCUs located in Gomma and Kersa had a negative percentage difference between number of DTP1 doses and the number of DTP3 doses administered, reflecting higher administration of DTP1 vaccines compared to DTP3. However, four out of nine PHCUs in Seka Chekorsa showed positive differences, a trend that was also seen for the comparison between ANC4 and ANC1 coverage, suggesting that there might be some issues with the quality of the reported data.

### External consistency of coverage rates

Most Pearson correlation coefficients suggested weak or moderate linear associations between HMIS and survey estimates in all three districts (Table 5). While strong correlation coefficients were observed for SBA and early PNC at the newborn level in Gomma (0.81 and 0.72, respectively), and for malaria in pregnancy (0.84), most ICC values were close to zero or negative, which demonstrates that the between-method variability (i.e. between HMIS and cross-sectional survey) and/or method error was significantly more important than the within-district variability. This suggests a large degree of discordance between the HMIS and the survey data.

**Table 5:** Pearson correlation coefficients and intraclass correlation coefficients for the relationship between HMIS and survey estimates from three districts in Jimma Zone, Ethiopia.

Pearson correlation coefficients							
	ANC1	ANC4	SBA	PNC (mothers)	PNC (newborns)	Stillbirth	Malaria in pregnancy^a^
**Gomma**	0.47	0.48	0.86	0.72	0.75	-0.29	0.84
**Seka Chekorsa**	-0.58	-0.02	0.37	0.65	0.41	0.48	
**Kersa**	-0.03	0.42	0.26	0.34	0.28	1.000	
**Intraclass correlation coefficients**							
**Gomma**	-0.02	0.17	0.26	0.15	0.02	-0.10	0.01
**Seka Chekorsa**	-0.54	-0.01	0.03	0.03	0.02	0.15	
**Kersa**	-0.03	0.24	0.04	-0.004	-0.01	-0.09	

ANC1- antenatal care firs visit, ANC4 –antenatal care fourth visit, SBA—skilled birth attendance, PNC—postnatal care, DTP1—Diphtheria, Tetanus and Pertussis first dose, DTP3—Diphtheria, Tetanus and Pertussis third dose

^a^ Data for this indicator are only available at the district level

The Bland-Altman analyses also indicate poor agreement between the HMIS and the cross-sectional survey estimates (Table 6 and S1–S4 Figs). In Gomma, the Bland-Altman analysis showed a negative systematic bias of -25.1% (95% CI: -40.9, -20.7%) for the proportion of women who attended ANC1, suggesting that, on average, ANC1 estimates in the HMIS were lower than estimates obtained from the survey. While the concordance between the two methods was relatively high for ANC4 in Gomma and Kersa, poor agreement was observed in Seka Chekorsa (median difference of 47.5% (95% CI: 32.6–73.5)). In all three districts, the HMIS reported higher SBA and PNC service uptake than the survey, while good agreement was observed in all three districts for stillbirth rates and rates of malaria infection during pregnancy.

**Table 6:** Bland-Altman summary statistics for the agreement analysis between HMIS and survey data from three districts in Jimma Zone, Ethiopia.

	Median difference (%) (95% CI)	95% limits of agreement (2.5^th^, 97.5^th^ percentiles)	Number of Potential Outliers
**ANC1**			
Gomma	-25.10 (-40.90, -20.70)	-65.60, -5.70	2
Kersa	-0.61 (-51.2, 19.11)	-51.20, 19.11	2
Seka Chekorsa	1.71 (-18.31, 41.32)	-18.31, 41.32	2
**ANC4**			
Gomma	-1.50 (-29.03, 8.10)	-32.91, 14.82	2
Kersa	15.16 (-16.16, 30.76)	-16.16, 30.76	1
Seka Chekorsa	47.55 (-32.61, 73.48)	-19.81, 73.48	3
**SBA**			
Gomma	-20.83 (-12.80, 29.36)	-3.93, 31.64	2
Kersa	39.61 (20.11, 59.05)	20.11, 59.05	2
Seka Chekorsa	63.74 (52.56, 79.85)	36.24, 79.85	2
**PNC (mothers)**			
Gomma	38.36 (29.37, 48.10)	9.40, 49.22	3
Kersa	54.01 (20.83, 67.17)	20.83, 67.17	2
Seka Chekorsa	72.54 (68.12, 89.68)	60.71, 89.68	3
**PNC (newborns)**			
Gomma	44.93 (15.23, 53.16)	15.23, 53.73	2
Kersa	55.26 (19.5, 68.08)	19.50, 68.08	2
Seka Chekorsa	75.09 (67.43, 93.72)	62.34, 93.72	3
**Stillbirth Rate**			
Gomma	-0.55 (-0.83, 0.46)	-2.01, 0.57	1
Kersa	0.15 (-0.67, 0.70)	-0.67, 0.70	2
Seka Chekorsa	0.00 (-0.75, 0.16)	-0.75, 0.16	2
**Malaria in Pregnancy**^a^			
	-1.42 (-1.98, -0.81)	-1.98, -0.81	0

ANC1- antenatal care firs visit, ANC4 –antenatal care fourth visit, SBA—skilled birth attendance, PNC—postnatal care, DTP1—Diphtheria, Tetanus and Pertussis first dose, DTP3—Diphtheria, Tetanus and Pertussis third dose, CI—confidence interval.

^a^ Data for this indicator are only available at the district level.

## Discussion

Using an established data quality assessment tool developed by the WHO, we identified several issues related to the quality of MCH data collected within the HMIS in Jimma Zone, Ethiopia. Many PHCUs failed to submit complete and timely reports to their respective district health office that reflected the monthly utilization of their MCH services. The observed rates of completeness and timeliness in all districts were lower than the 90% limit sets by the FMoH [18]. The failure to submit reports, and thus MCH coverage estimates, to the next reporting level may result in a partial and incomplete representation of MCH service provision at the district level. This could have important implications for the health of pregnant women and newborns living in these districts, as information reported by the PHCUs is used by the district health offices to guide future plans. Similar challenges with the completeness and timeliness of HMIS reporting have been observed in other studies [18,27,28].

Our assessment of the consistency between indicators revealed potential gaps that warrant further investigation, as well as potential data quality issues. For example, our observation that ANC1 was higher than DPT1 coverage in some PHCUs suggests that more women may be attending antenatal care compared to infants receiving DPT vaccination, or this may reflect issues in data quality. However, this discrepancy may also reflect a higher number of pregnancies than live births, which was not assessed directly. The finding that DPT3 was higher than DPT1 in several PHCUs suggests that many infants who received their first dose may not have received their third one, an issue that warrants further investigation. Higher DPT3 than DPT1 coverage estimates, and ANC4 than ANC1 coverage estimates, are indicative of data quality issues. The latter may also reflect double counting of women at multiple levels of the health system if these discrepancies are not identified and investigated at the PHCU and district levels, and corrected to ensure data quality [29].

Comparison of HMIS data with cross-sectional population-representative survey data also revealed data quality issues, suggesting that some PHCUs and district health offices may over-report certain MCH indicators, including SBA and PNC. This is consistent with a national evaluation conducted in 2016, which identified that over-reporting was important, with 43% of PHCUs in Oromiya region over-reporting ANC attendance by 10% or more, while both under- and over-reporting of SBA data were observed [15]. Over-representing the proportion of women attending health services may have a significant impact on women’s health outcomes if they are not attending these services, and resources may not be going towards improving their access to these essential services.

Although we conducted this evaluation using an established data quality tool and rigorous statistical methods, there are several limitations that constrained our findings. First, we did not perform an exhaustive assessment of all MCH indicators collected in the HMIS. Due to time constraints, indicators related to the use of family planning methods or to the prevention of mother-to-child transmission of HIV were not considered, despite the importance given to them within PHCUs’ service reports [21]. We also did not assess the consistency between information contained in source documents (i.e. health facility registers and district health office summary reports) and the information reported in the HMIS, thereby precluding our assessment of the levels at which most reporting errors occur. Our study is also limited by the fact that we did not consider private health facilities in our assessments. This was mainly due to time constraints, but also due to private health facilities being less likely to provide the common MCH services that are reported to district health offices [21], and only infrequently reporting data on their health service attendance. Focusing on the quality of MCH services data provided in governmental health facilities thus provides an adequate assessment of the quality of MCH data in Jimma Zone. Despite these limitations, a particular strength of this study was the comparison with data from a large household-based cross-sectional survey that recruited women attending the PHCUs of interest, allowing us to perform an agreement analysis. While self-reporting and interviewer biases may have occurred in administering the survey, comprehensive training of survey interviewers was used to limit such bias.

Our findings support those of previous studies, which suggest that strategies to improve the quality of MCH data collected within the HMIS are needed [17]. Important strategies may focus on ensuring effective communication processes between health workers, HMIS supervisors at the health facility level, and district-level officials [17]. Furthermore, given their situation at the primary level of health data collection, there is an urgent need to ensure that health post and health centre workers are well trained in data reporting and the performance of data quality checks. Additional qualitative research on the perceptions of health workers and HMIS officers roles in the management and sustainability of the HMIS would be beneficial to understand their concerns and their ideas about how they can contribute to enhancing the quality of the data gathered within the HMIS.

It is also important to note that barriers other than those relating to the lack of training and knowledge of health workers and supervisors may explain the poor quality of MCH data in the HMIS. While the introduction of an electronic HMIS has resulted in improvements in the reporting, monitoring and evaluation of health data, the availability of other essential material resources to perform key HMIS tasks may be more limited. In other regions of Ethiopia it has been noted that PHCUs and district health offices sometimes lack access to computers to facilitate the compilation of the data and its utilization [20]. Belay et al. also noted that HEWs in health posts may not have access to calculators to perform basic calculations to assess the use of services in their catchment areas, which can limit their ability to report accurate data to higher levels of the health system, as well as a shortage of registration and summary forms [20]. Overcoming material barriers can serve to strengthen HMIS data for informing MCH and other programmatic health efforts in Ethiopia.

## Supporting information

S1 Appendix Survey questions used in HMIS data quality assessment for comparison of MCH coverage estimates. (DOCX)

S1 Fig Bland-Altman plots for the agreement of malaria in pregnancy rate estimates between the HMIS and the cross-sectional survey in three district of Jimma Zone, Ethiopia. (DOCX)

S2 Fig Bland-Altman plots for the agreement of maternal and child health indicator coverage estimates between the HMIS and the survey in Gomma. (DOCX)

S3 Fig Bland-Altman plots for the agreement of maternal and child health indicator coverage estimates between the HMIS and the survey in Kersa. (DOCX)

S4 Fig Bland-Altman plots for the agreement of maternal and child health indicator coverage estimates between the HMIS and the survey in Seka Chekorsa. (DOCX)
