# Supplementary material for: A quality assessment of Health Management Information System (HMIS) data for maternal and child health in Jimma Zone, Ethiopia
Source: PLoS One. 2019 Mar 11;14(3):e0213600. doi: 10.1371/journal.pone.0213600 (PMC6411115; doi:10.1371/journal.pone.0213600)
Supplement: S1 Appendix — (DOCX) [file pone.0213600.s001.docx]

**S1 Appendix. Survey questions used in the HMIS data quality assessment for comparison of maternal health services coverage estimates.**

**Antenatal care**

|  | Did you see anyone for antenatal care during your **last pregnancy**? | ^1^⭘ Yes | ^0^⭘ No | |
| --- | --- | --- | --- | --- |
|  | How many times did you visit the health facility for antenatal care? | ⬜ ⬜ | | ^00^⭘ Do not remember |
|  | During your **last pregnancy**, how many months pregnant were you when you went for your **first** antenatal care visit at the health facility? | ⬜ ⬜ months | | ^00^⭘ Do not remember |
|  | How many months pregnant were you when you **last** received antenatal care for your last pregnancy? | ⬜ ⬜ months | | ^00^⭘ Do not remember |
|  | Where did you **mainly** receive antenatal care from during your last pregnancy?  INTERVIEWER: Select the place woman went most often for ANC care. Enter all sites mentioned in “Other” if it is not clear which site was used most often. | ^1^⭘ Own home  ^2^⭘ Someone’s home  ^3^⭘ Government hospital  ^4^⭘ Government health centre | | ^5^⭘ Government health post  ^6^⭘ Private hospital  ^7^⭘ Private clinic  ^88^⭘ Other (*Specify*) |

**Intrapartum care**

|  | Where did you give birth to your last child? | ^1^⭘ Own home  ^2^⭘ Someone else’s home (ex: relative, TBA)  ^3^⭘ Government hospital  ^4^⭘ Government health centre  ^5^⭘ Government health post | ^6^⭘ Private hospital  ^6^⭘ Private clinic  ^88^⭘ Other (*Specify*) |
| --- | --- | --- | --- |
|  | Who assisted with the delivery of your last child? | ^1^⭘ Doctor  ^2^⭘ Nurse/midwife  ^3^⭘ TBA  ^4^⭘ Relative/friend | ^5^⭘ HEW  ^88^⭘ Other (*Specify*) |

**Postpartum care**

|  | After you gave birth to your last child, did someone check on your health?  For example someone examining you or asking you questions about your health? | ^1^⭘ Yes | ^0^⭘ No |
| --- | --- | --- | --- |
|  | How long after delivery did the **first** check take place? | ⬜ ⬜ hours  ⬜ ⬜ days | ⬜ ⬜ weeks  ^98^⭘ Do not remember |
|  | Who checked on your health at that time? | ^1^⭘ Doctor  ^2^⭘ Nurse/midwife  ^3^⭘ TBA  ^4^⭘ Relative/friend | ^5^⭘ HEW  ^88^⭘ Other (*Specify*) |
|  | Where did this **first** check up take place? | ^1^⭘ Own home  ^2^⭘ Someone else’s home (ex: relative, TBA)  ^3^⭘ Government hospital  ^4^⭘ Government health centre  ^5^⭘ Government health post | ^6^⭘ Private hospital  ^6^⭘ Private clinic  ^88^⭘ Other (*Specify*) |
|  | Did anyone check on **your baby’s** health?  For example, look at the cord to see if it was ok? | ^1^⭘ Yes | ^0^⭘ No |
|  | How long after delivery did the **first** check on your baby take place? | ⬜ ⬜ hours  ⬜ ⬜ days | ⬜ ⬜ weeks  ^98^⭘ Do not remember |
|  | Who checked on the baby’s health at that time? | ^1^⭘ Doctor  ^2^⭘ Nurse/midwife  ^3^⭘ TBA  ^4^⭘ Relative/friend | ^5^⭘ HEW  ^88^⭘ Other (*Specify*) |
|  | Where did this first check on the baby’s health take place? | ^1^⭘ Own home  ^2^⭘ Someone else’s home (ex: relative, TBA)  ^3^⭘ Government hospital  ^4^⭘ Government health centre  ^5^⭘ Government health post | ^6^⭘ Private hospital  ^6^⭘ Private clinic  ^88^⭘ Other (*Specify*) |
