# Supplementary material for: A quality assessment of Health Management Information System (HMIS) data for maternal and child health in Jimma Zone, Ethiopia
Source: PLoS One. 2019 Mar 11;14(3):e0213600. doi: 10.1371/journal.pone.0213600 (PMC6411115; doi:10.1371/journal.pone.0213600)

**S1 Fig. Bland-Altman plots for the agreement of malaria in pregnancy rate estimates between the HMIS and the cross-sectional survey in three district of Jimma Zone, Ethiopia**

To maintain the confidentiality of the districts on this plot, a letter was assigned to each district (A – C). In certain cases, the upper limit of agreement coincides with the upper 95% CI limit of the median, and hence only one limit is represented. Abbreviations: CI - confidence interval; diff - difference


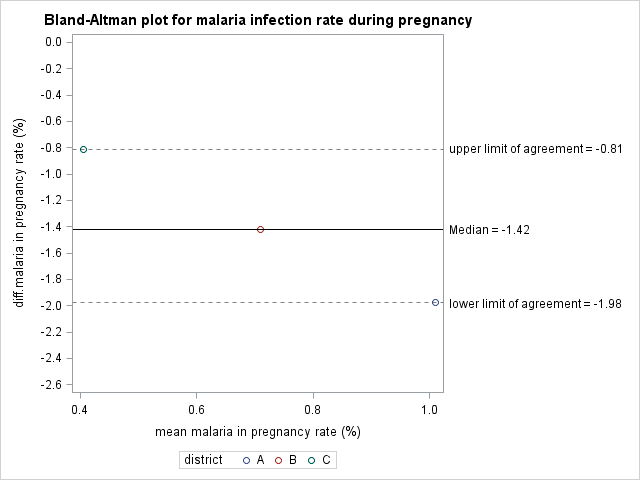

Supplement: S1 Fig — (DOCX) [file pone.0213600.s002.docx]
