# Supplementary material for: A quality assessment of Health Management Information System (HMIS) data for maternal and child health in Jimma Zone, Ethiopia
Source: PLoS One. 2019 Mar 11;14(3):e0213600. doi: 10.1371/journal.pone.0213600 (PMC6411115; doi:10.1371/journal.pone.0213600)

**S4 Fig. Bland-Altman plots for the agreement of maternal and child health indicator coverage estimates between the HMIS and the survey in Seka Chekorsa**

To maintain the confidentiality of the health facilities, a letter was assigned to each health facility (A – H). In certain cases, the upper limit of agreement coincides with the upper 95% CI limit of the median, and hence only one limit is represented. Abbreviations: CI - confidence interval; diff - difference

**S4A Fig. Bland-Atman plot for Antenatal Care 1+ Coverage in Seka Chekorsa**


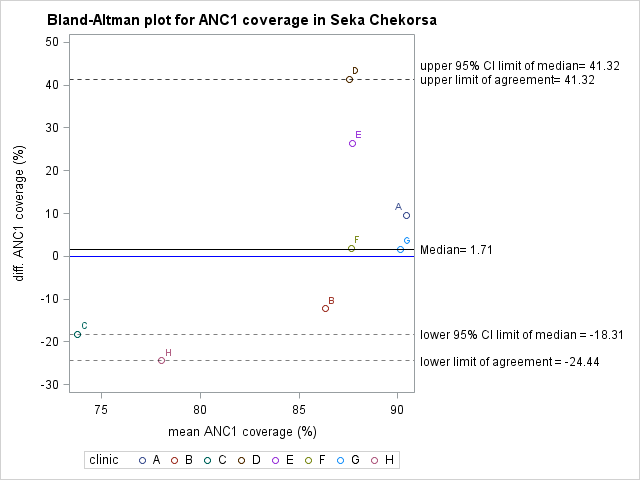


**S4B Fig. Bland-Atman plot for Antenatal Care 4+ Coverage in Seka Chekorsa**


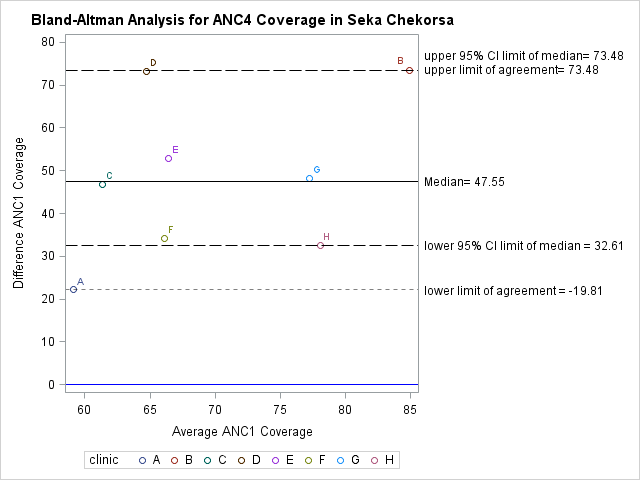


**S4C Fig. Bland-Atman plot for Skilled Birth Attendance Coverage in Seka Chekorsa**


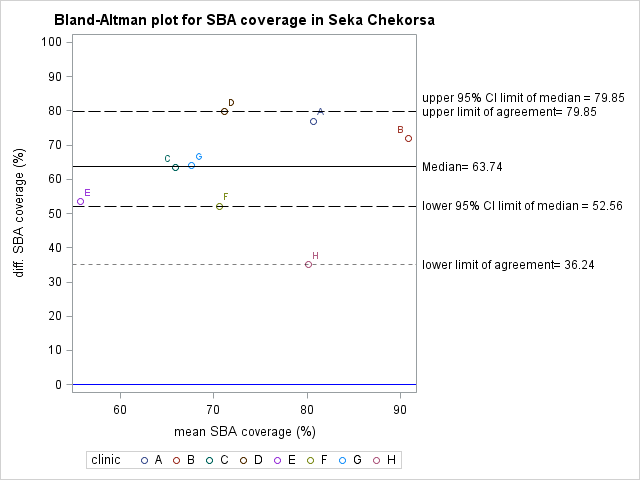


**S4D Fig. Bland-Atman plot for Postnatal Care of the Mother Coverage in Seka Chekorsa**


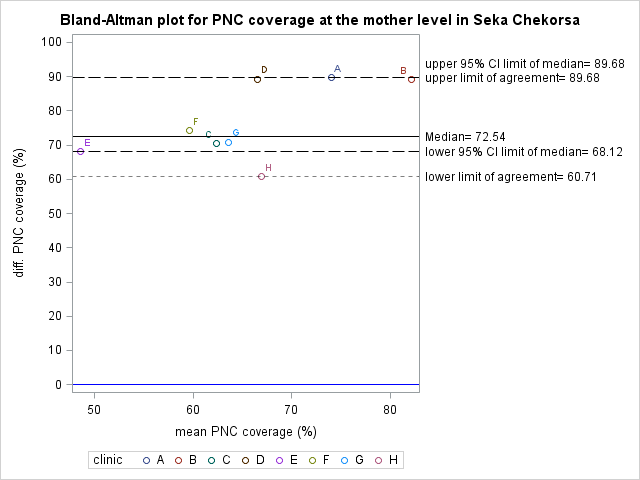


**S4E Fig. Bland-Atman plot for Postnatal Care of the Newborn Coverage in Seka Chekorsa**


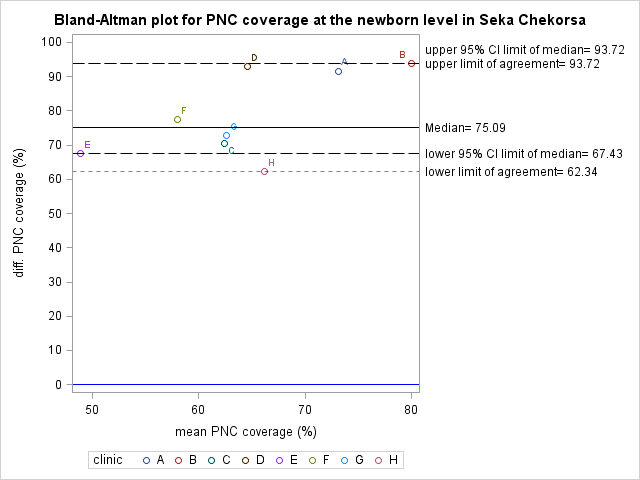


**S4F Fig. Bland-Atman plot for Stillbirth Rate in Seka Chekorsa**


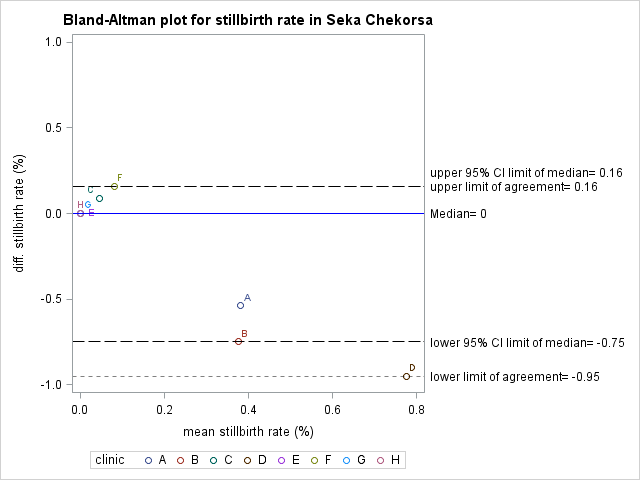

Supplement: S4 Fig — (DOCX) [file pone.0213600.s005.docx]
